# Empagliflozin to prevent progressive adverse remodelling after myocardial infarction (EMPRESS‐MI): rationale and design

**DOI:** 10.1002/ehf2.14830

**Published:** 2024-05-07

**Authors:** Jaclyn Carberry, Mark C. Petrie, Matthew M.Y. Lee, Katriona Brooksbank, Ross T. Campbell, Richard Good, Pardeep S. Jhund, Peter Kellman, Ninian N. Lang, Kenneth Mangion, Patrick B. Mark, Alex McConnachie, John J.V. McMurray, Barbara Meyer, Vanessa Orchard, Aadil Shaukat, Stuart Watkins, Paul Welsh, Naveed Sattar, Colin Berry, Kieran F. Docherty

**Affiliations:** ^1^ British Heart Foundation Glasgow Cardiovascular Research Centre University of Glasgow Glasgow UK; ^2^ Golden Jubilee National Hospital Clydebank UK; ^3^ West of Scotland Innovation Hub, National Health Service Glasgow UK; ^4^ National Heart, Lung, and Blood Institute National Institutes of Health Bethesda MD USA; ^5^ Robertson Centre for Biostatistics, School of Health and Wellbeing University of Glasgow Glasgow UK

**Keywords:** Heart failure, Myocardial infarction, SGLT2 inhibitor

## Abstract

**Aims:**

Patients with a reduced left ventricular ejection fraction (LVEF) following an acute myocardial infarction (MI) are at risk of progressive adverse cardiac remodelling that can lead to the development of heart failure and death. The early addition of a sodium‐glucose cotransporter 2 (SGLT2) inhibitor to standard treatment may delay or prevent progressive adverse remodelling in these patients.

**Methods and results:**

EMpagliflozin to PREvent worSening of left ventricular volumes and Systolic function after Myocardial Infarction (EMPRESS‐MI) is a randomized, double‐blind, placebo‐controlled, multi‐centre trial designed to assess the effect of empagliflozin on cardiac remodelling evaluated using cardiovascular magnetic resonance (CMR) in 100 patients with left ventricular systolic dysfunction following MI. Eligible patients were those ≥12 h and ≤14 days following acute MI, with an LVEF <45% by CMR. Patients were randomized to empagliflozin 10 mg once a day or matching placebo. The primary outcome will be change in left ventricular end‐systolic volume indexed to body surface area over 24 weeks from randomization. Secondary endpoints include measures of left ventricular and atrial volumes, left ventricular mass, LVEF, and circulating cardiac biomarkers.

**Conclusions:**

EMPRESS‐MI will assess the effect of the SGLT2 inhibitor empagliflozin on cardiac remodelling in patients with left ventricular systolic dysfunction after an acute MI.

## Introduction

The widespread utilization of emergency reperfusion therapy and advances in pharmacological secondary prevention have substantially improved survival rates following an acute myocardial infarction (MI).^1^, ^2^ However, some patients still develop heart failure in the weeks, months, and years that follow.^2^ As the development of heart failure is associated with substantial morbidity and mortality, its prevention is an important therapeutic goal.^3^

The pathological precursor to heart failure with reduced ejection fraction (HFrEF) after MI is a reduction in systolic function and progressive ventricular dilatation.^4^ A key advance in the treatment of MI was the demonstration that pharmacological attenuation of adverse remodelling was associated with a reduction in the subsequent risk of heart failure and mortality. By inhibiting the maladaptive neurohumoral activation that promotes adverse remodelling, angiotensin‐converting enzyme inhibitors or angiotensin receptor blockers, beta‐blockers, and mineralocorticoid receptor antagonists reduce the risk of heart failure and mortality following MI.^4^, ^5^, ^6^, ^7^, ^8^, ^9^, ^10^, ^11^, ^12^, ^13^, ^14^, ^15^ Recently, augmenting cardioprotective natriuretic peptides with a neprilysin inhibitor did not reduce the risk of incident heart failure or cardiovascular mortality in high‐risk patients following MI.^16^ Novel therapeutic options that provide additive benefits to neurohumoral inhibition are therefore required.

Sodium‐glucose cotransporter 2 (SGLT2) inhibitors reduce the risk of worsening heart failure and mortality in patients with chronic heart failure across the full spectrum of left ventricular ejection fraction (LVEF).^17^, ^18^ They also prevent the development of heart failure in patients with type 2 diabetes mellitus (T2DM) and established atherosclerotic cardiovascular disease, many of whom have a history of MI.^19^ Multiple mechanisms underlying the clinical benefits of SGLT2 inhibitors have been suggested and include a beneficial remodelling effect when added to neurohumoral modulating therapies in patients with established HFrEF.^20^, ^21^, ^22^ Recently, the SGLT2 inhibitor empagliflozin did not significantly reduce the risk of heart failure hospitalization or death from any‐cause in high‐risk patients following acute MI.^23^ However, exploratory analyses suggested that empagliflozin reduced the risk of the development of heart failure.^24^ Whether this benefit is related to a remodelling benefit of SGLT2 inhibitors in high‐risk patients with left ventricular systolic dysfunction following MI is unknown.

Consequently, we designed the EMpagliflozin to PREvent worSening of left ventricular volumes and Systolic function after Myocardial Infarction (EMPRESS‐MI) randomized, placebo‐controlled trial to test the hypothesis that the SGLT2 inhibitor empagliflozin, in addition to current evidence‐based therapy, will attenuate adverse left ventricular remodelling measured using cardiovascular magnetic resonance (CMR) imaging in high‐risk patients following MI.

## Methods

### Trial organization and sources of funding

EMPRESS‐MI was conceived and designed by the Trial Steering Committee. The trial was co‐sponsored by the University of Glasgow and the National Health Service (NHS) Greater Glasgow and Clyde Health Board and registered at ClinicalTrials.gov (NCT05020704). The lead site was the NHS Golden Jubilee National Hospital. Funding for the trial and provision of empagliflozin and a matching placebo was provided by Boehringer Ingelheim. Boehringer Ingelheim had no role in the trial design, trial conduct, and will not be involved in data analysis or interpretation. The trial protocol and any substantial amendments were approved by the Newcastle Research Ethics Committee (22/NE/0030). The first patient was randomized on the 6 October 2022. The last patient last visit is anticipated to take place in June 2024.

### Patient eligibility

Eligible patients were men or women ≥18 years of age, who had a type 1 MI, that is, an ST‐elevation MI (STEMI) or non‐STEMI (according to the fourth universal definition of MI), and an LVEF ≤40% measured by echocardiography.^25^ Key exclusion criteria were a history of HFrEF, contraindications to SGLT2 inhibitors (a history of type 1 diabetes mellitus, ketoacidosis, allergy to SGLT2 inhibitors, planned or current use of SGLT2 inhibitors or active genital infection). Patients with radiological contrast allergy or estimated glomerular filtration rate (eGFR) < 30 mL/min/1.73 m^2^ (measured using the modification of diet in renal disease [MDRD] formula) were also excluded. Patients with permanent or persistent atrial fibrillation or an implanted cardiac device were excluded to avoid potential CMR image degradation. The complete inclusion and exclusion criteria are listed in *Table* *1*. Participants were randomized after at least 12 h and before 14 days following admission with MI.

**Table 1:** Inclusion and exclusion criteria for the EMPRESS‐MI trial

Inclusion criteria
Male or female ≥18 years of age
Informed consent
Diagnosis of a type 1 acute myocardial infarction meeting the Fourth Universal Definition of Myocardial Infarction (ST elevation myocardial infarction or non‐ST elevation myocardial infarction)
Left ventricular ejection fraction <45% as measured by CMR performed ≥12 h and ≤14 days following hospital admission with an acute type 1 myocardial infarction. For patients with an in‐hospital myocardial infarction as qualifying event, randomization must still occur within 14 days of hospital admission
eGFR ≥30 mL/min/1.73 m^2^ at the time of randomization (calculated using the MDRD formula)
Exclusion criteria
Inability to give informed consent, for example, due to significant cognitive impairment
Diagnosis of chronic heart failure with reduced ejection fraction prior to admission with acute myocardial infarction
Systolic blood pressure <90 mmHg at randomization measured after 5 min in a supine or sitting position
Cardiogenic shock or use of intravenous inotropes in last 24 h before randomization
Coronary artery bypass graft planned at time of randomization
Type II acute myocardial infarction
Any current severe (stenotic) valvular heart disease
Diagnosis of Takotsubo cardiomyopathy
Type I diabetes mellitus
History of ketoacidosis
Pacemaker, implantable cardioverter defibrillator, or cardiac resynchronization therapy device
Permanent or persistent atrial fibrillation
Enrolment in another randomized clinical trial involving medical or device‐based interventions (co‐enrolment in observational studies is permitted)
Currently pregnant, planning pregnancy, or currently breastfeeding
History of allergy to SGLT2 inhibitor
Current or planned use of an SGLT2 inhibitor at time of randomization
Active genital tract infections
Anyone who, in the investigators' opinion, is not suitable to participate in the trial for other reason
Contra‐indication to contrast‐enhanced CMR, that is, claustrophobia, metallic foreign object unsuitable for CMR

CMR, cardiovascular magnetic resonance; eGFR, estimated glomerular filtration rate; MDRD, modification of diet in renal disease; SGLT2, sodium‐glucose cotransporter 2.

### Screening

Admission transthoracic echocardiograms, performed ≥12 h from admission as part of routine clinical care, were reviewed to identify patients with LVEF ≤40%, measured by Simpson's Biplane or estimated by visual assessment. Those who met the inclusion and exclusion criteria were approached for consent. Consenting patients then had a baseline CMR scan, which included kidney imaging. Patients with an LVEF <45% by CMR proceeded to randomization (changed from an LVEF ≤40% by an amendment to the trial protocol on 23 February 2023), and those with LVEF ≥45% were excluded. Patients who initially failed screening could be re‐screened once more for reasons other than not meeting the LVEF criterion.

### Trial procedures

#### Randomization and blinding

Patients were randomized in a 1:1 ratio to receive either empagliflozin 10 mg once a day, or matched placebo. The randomization schedule was computer‐generated by the method of randomized permuted blocks, with random block lengths of 4 and 6. Randomization was stratified by CMR‐measured left ventricular end‐systolic volume indexed to body surface area (LVESVI) (≤45 mL/m^2^/>45 mL/m^2^), the prescription of loop diuretics at the time of randomization, and the presence of T2DM (either an established diagnosis or a glycated haemoglobin [HbA1c] ≥ 48 mmol/mol at the index admission). Before randomization, baseline assessments were performed, including measurement of weight and height, a physical examination, a 12‐lead electrocardiogram, a EuroQol 5‐Dimension 5‐Level (EQ‐5D‐5L) questionnaire, standard laboratory blood tests including kidney function, liver function, HbA1c and a full blood count, and blood and urine were collected for biomarker analysis. Randomization and administration of the first dose of trial drug took place within 24 h of the baseline CMR. All participants and trial staff were blinded to treatment allocation.

#### Follow‐up visits

**Figure 1:**
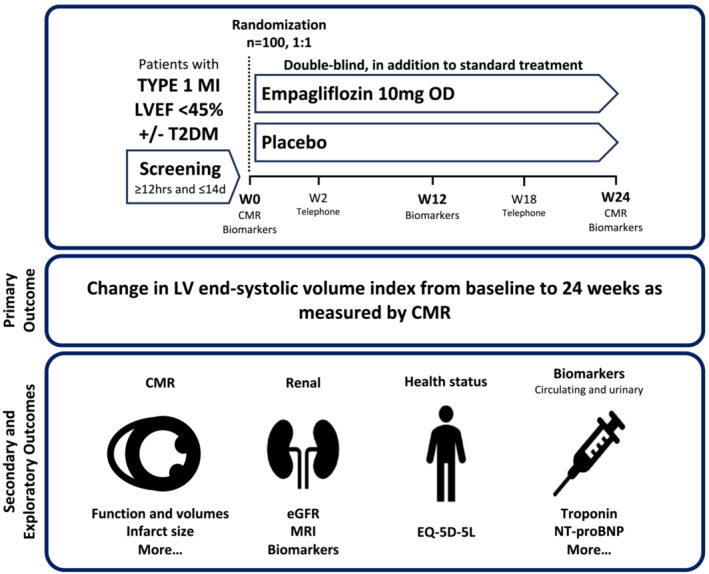
EMPRESS‐MI trial design. EMPRESS‐MI trial design. One hundred patients with type 1 myocardial infarction and left ventricular ejection fraction <45% will be randomized on a 1:1 basis to empagliflozin once a day or placebo for 24 weeks. The primary outcome is the change in left ventricular end‐systolic volume indexed to body surface area from baseline to 24 weeks as measured by cardiovascular magnetic resonance imaging. CMR, cardiovascular magnetic resonance; eGFR, estimated glomerular filtration rate; EQ‐5D‐5L, EuroQol 5‐Dimension 5‐Level; LV, left ventricular; LVEDVI, left ventricular end‐diastolic volume index; LVEF, left ventricular ejection fraction; MI, myocardial infarction; NT‐proBNP, N‐terminal pro‐B‐type natriuretic peptide; OD, once daily.

Following randomization, trial visits took place at 2, 12, 18, and 24 weeks. The visit schedule is outlined in *Figure* *1*. To mitigate any potential coronavirus pandemic restrictions, visit 2 (at 2 weeks) and visit 4 (at 18 weeks) were carried out by a telephone call. All other visits were face‐to‐face.

#### Cardiovascular magnetic resonance imaging

CMR was performed at baseline and 24 weeks following randomization on a single 1.5 Tesla Siemens MAGNETOM Avanto scanner with a 12‐element phased array cardiac surface coil and a 12‐element phased array body surface coil for kidney imaging.

**Figure 2:**
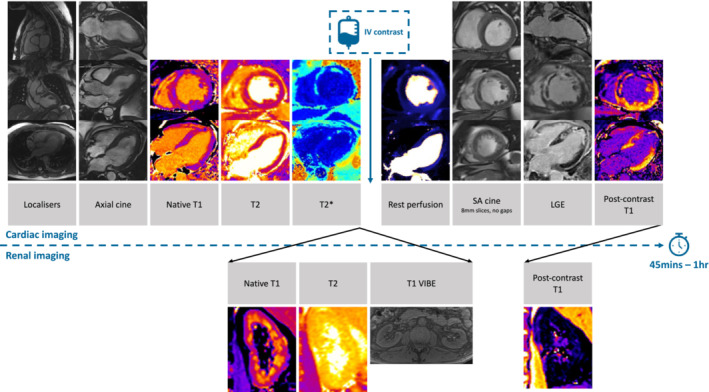
Cardiovascular magnetic resonance imaging protocol outline. Cardiovascular magnetic resonance imaging will be performed pre‐randomization and at 24 weeks on a single 1.5 Tesla Siemens MAGNETOM Avanto scanner. IV, intravenous; LGE, late gadolinium enhancement; SA, short‐axis; VIBE, volumetric interpolated breath‐hold examination.

The imaging protocol is outlined in *Figure* *2* and included steady state free procession (SSFP) sequencing to acquire left ventricular axial cine images (2‐, 3‐, and 4‐chamber) and a short‐axis cine stack from pulmonary veins to left ventricular apex (8 mm slices, no gaps). Native and post‐contrast T1 mapping of the 4‐chamber and mid short‐axis slice were acquired using a modified Look‐Locker inversion‐recovery sequence. T2 mapping of the 4‐chamber and mid short‐axis was acquired using an SSFP sequence. T2* mapping of the 4‐chamber and mid short‐axis was acquired using gradient echo acquisition and free‐breathing motion corrected technique. Rest perfusion imaging of the 4‐chamber and three short‐axis slices (base, mid and apex), was acquired following intravenous injection of half‐dose gadolinium‐based contrast (Gadovist 0.05 mmol/kg 4 mL/s with 30 mL saline flush). Full dose contrast was then administered (Gadovist 0.1 mmol/kg at 4 mL/s with 30 mL saline flush) 10–15 min before late gadolinium enhancement imaging (3 long‐axis views, and short‐axis stack) was acquired using phase‐sensitive inversion recovery pulse sequence and free‐breathing motion corrected technique. T1 modified Look‐Locker inversion recovery pre‐ and post‐contrast and T2 fast low‐angle shot (FLASH) coronal imaging of the kidneys were acquired. T1 volumetric interpolated breath‐hold examination FLASH imaging in the axial plane of the kidneys were also acquired.

All scans were reviewed by a single cardiologist for the purpose of generating a clinical report. Scans were then pseudo‐anonymized and analysed by a single operator in accordance with the Society for Cardiovascular Magnetic Resonance and European Society of Cardiovascular Imaging guidelines for reporting CMR examinations.^26^, ^27^ The baseline and 24‐week scans were analysed in pairs to reduce intra‐observer variability, and the operator was blinded to treatment allocation. A random selection of scans (10%) was analysed by a second operator blinded to treatment assignment for assessment of inter‐operator variability and quality assurance.

#### Biomarkers

Venous blood and urine samples were collected at baseline, 12 weeks and 24 weeks following randomization, for biomarker analysis. Samples were collected and immediately chilled, then centrifuged as soon as possible at 1500 *g* at 4°C for 10 min. Samples were then aliquoted and stored at −80°C. After completion of the trial, all samples were transferred to the central laboratory (University of Glasgow) for batch analysis.

#### Patient‐reported health status

Patients were asked to complete an EQ‐5D‐5L questionnaire at baseline and 24 weeks after randomization to assess the effect of empagliflozin on quality of life.

#### Development of heart failure and study drug discontinuation during the trial

If patients developed heart failure during the trial, they were offered an open‐label SGLT2 inhibitor according to clinical practice guidelines.^28^ Patients who started open‐label SGLT2 inhibitor or withdrew from the trial after 12 weeks were asked to undergo an ‘end‐of‐trial’ CMR scan.

### Trial outcomes

The primary, secondary and exploratory outcomes will be measured as change from baseline to 24 weeks of follow‐up. The between‐treatment differences in these changes will be analysed. All CMR measurements will be indexed to body surface area, measured at the time of the scan using the Mosteller formula.

#### Primary outcome

The primary outcome is the change in LVESVI measured by CMR.

#### Secondary outcome

The secondary outcomes are as follows:

- Change in other CMR measures of cardiac remodelling:
  - left ventricular end‐diastolic volume indexed to body surface area (LVEDVI);
  - LVEF;
  - left atrial volume indexed to body surface area (LAVI);
  - left ventricular mass indexed to body surface area;
  - infarct size—measured in mass and as a percentage of myocardium with signal intensity over 5 standard deviations from remote myocardium.
- Change in N‐terminal pro‐B‐type natriuretic peptide (NT‐proBNP).
- Change in high‐sensitivity troponin I.

#### Exploratory outcomes

The exploratory outcomes are as follows:

- Change in other CMR biomarkers of adverse remodelling: including left ventricular circumferential strain using feature‐tracking, extracellular volume in the remote and infarct regions, microvascular obstruction, myocardial haemorrhage, myocardial salvage index, remote zone native T1, right ventricular volumes and ejection fraction, resting myocardial blood flow and cardiac biomechanics derived from mathematical modelling.
- The proportion of patients in remodelling groups at 24 weeks, defined as; group 1 reverse left ventricular remodelling [≥12% decrease in LVESVI]; group 2: no left ventricular remodelling [changes in LVEDVI and LVESVI <12%]; group 3: adverse left ventricular remodelling with compensation [≥12% increase in LVEDVI only]; and group 4: adverse left ventricular remodelling [≥12% increase in both LVESVI and LVEDVI]^29^).
- Change in circulating and urinary biomarkers reflecting neurohumoral activation, kidney function, tissue remodellling, inflammation, haematopoiesis (including ferritin, hepcidin, and iron), and other pathways relevant to the underlying conditions of the patients and the actions of empagliflozin (including HbA1c and uric acid).
- Change in patient‐reported health status measured using EQ‐5D‐5L questionnaire.
- Change in circulating biomarkers of kidney function and injury including serum creatinine and eGFR (MDRD formula).
- Change in kidney native T1 relaxation time and T2 relaxation time.

### Statistical considerations

Using data from recent trials, we calculated that 50 patients in each treatment group would provide >90% power (α level = 0.05) to detect a mean between‐group difference in change in LVESVI from baseline of 6 mL/m^2^ (standard deviation of change = 7.8 mL/m^2^).^30^ We estimated 120 screened patients would allow for a 10% screen failure rate for LVEF and a 10% drop out rate for loss to follow‐up and death.

Analyses will be performed on an intention‐to‐treat basis. The primary analysis will compare the mean change in LVESVI in each randomized group using a linear regression model adjusted for baseline LVESVI, the use of a diuretic at baseline and the presence or not of T2DM. Similar methods will be applied to the secondary and exploratory outcomes. The effect of treatment will be presented with 95% confidence intervals (CI) and two‐sided *P*‐values. A detailed statistical analysis plan will be finalized prior to database lock and unblinding of randomized groups.

## Discussion

EMPRESS‐MI is a multi‐centre, randomized, double‐blind, placebo‐controlled trial designed to evaluate the effect of empagliflozin on cardiac remodelling over 24 weeks in patients with an LVEF <45% by CMR following an acute MI, with or without T2DM. The results of EMPRESS‐MI will provide novel multi‐parametric information on the potential additive benefit of SGLT2 inhibition to standard treatment in patients at high risk of developing heart failure.

### Evidence for an anti‐remodelling effect of sodium‐glucose cotransporter 2 inhibitors

Both the progression of existing HFrEF and the development of heart failure following an MI are driven by the process of adverse left ventricular remodelling. In light of this, neurohormonal inhibitors, such as beta‐blockers and renin angiotensin aldosterone system inhibitors, are recommended for the treatment of both conditions. These drugs improve outcomes in patients with established HFrEF, and also have a favourable effect on remodelling.^31^, ^32^, ^33^, ^34^, ^35^, ^36^, ^37^, ^38^ Subsequently, these therapies have also exhibited a similar beneficial effect in patients with post‐MI systolic dysfunction including a prevention of progressive adverse remodelling and the development of HFrEF.^4^, ^5^, ^6^, ^7^, ^8^, ^9^, ^10^, ^11^, ^12^, ^13^, ^14^ The necessity to test evidence‐based medical therapy for chronic HF in the setting of acute MI is illustrated by the Angiotensin Receptor–Neprilysin Inhibition in Acute Myocardial Infarction (PARADISE‐MI) trial, in which sacubitril/valsartan did not reduce the risk of the primary endpoint of cardiovascular death or incident heart failure in high‐risk patients following acute MI.^16^ The clinical scenario early after acute MI is very different to that in chronic HFrEF, with a sudden reduction in LVEF (that may only be transient due to myocardial ‘stunning’), emergency coronary intervention and reperfusion, and the concurrent initiation of several secondary prevention drugs. Therefore, it is essential to establish both the efficacy and safety of novel therapeutics in the setting of acute MI, and not simply translate evidence from chronic HFrEF.

SGLT2 inhibitors improve outcomes in HFrEF, and have an anti‐remodelling effect. Three randomized, placebo‐controlled trials have reported a consistent benefit of SGLT2 inhibitors on reducing left ventricular volumes in patients with established chronic HFrEF.^20^, ^21^, ^22^ Investigating the efficacy of SGLT2 inhibitors with regards to preventing adverse remodelling in this dedicated post‐MI trial will therefore provide novel, valuable information for the role of this class of drug as an additional secondary preventative therapy in this high‐risk population.

### Evidence for sodium‐glucose cotransporter 2 inhibitors after myocardial infarction

All of the large outcome trials of SGLT2 inhibitors in patients with T2DM, heart failure and chronic kidney disease excluded patients who were <8–12 weeks following acute MI. However, there are now two large outcome trials of SGLT2 inhibitors in patients following an acute MI, Empagliflozin in Patients Post Myocardial Infarction (EMPACT‐MI), and Dapagliflozin Effects on Cardiovascular Events in Patients With an Acute Heart Attack (DAPA‐MI).^23^, ^39^

The EMPACT‐MI trial randomized 6522 patients within 14 days of acute MI to empagliflozin 10 mg once daily or placebo.^40^ The inclusion criteria were an LVEF <45% and/or signs or symptoms of congestion, as well as at least one additional risk factor known to be associated with the risk of development of heart failure and mortality, including T2DM. The primary outcome was a composite of hospitalization for heart failure and all‐cause mortality, assessed in a time‐to‐first‐event analysis. The primary outcome did not differ significantly between the treatment groups during a median follow‐up of 17.9 months (hazard ratio [HR] 0.90; 95% CI 0.76 to 1.06; *P* = 0.21). However, in an exploratory analysis, empagliflozin reduced the risk of time‐to‐first heart failure hospitalization (HR 0.77; 95% CI 0.60 to 0.98; *P* = 0.031) and investigator‐reported adverse events of heart failure including those managed as an outpatient.^24^ The results were consistent across major patient subgroups, and demonstrate the potential role of empagliflozin in addition to guideline therapy for preventing heart failure hospitalizations in patients who are at high‐risk post‐MI.^23^

The DAPA‐MI trial randomized 4017 patients without diabetes following an acute MI with left ventricular systolic dysfunction and/or electrocardiographic evidence of a Q‐wave MI to dapagliflozin 10 mg once daily or placebo.^41^ The original primary outcome of DAPA‐MI was a composite of time to first cardiovascular death or heart failure hospitalization. However, due to a lower than expected event rate during the trial, this outcome was expanded prior to database lock to include non‐fatal MI, the occurance of atrial fibrillation/flutter, new‐onset diabetes, New York Heart Association (NYHA) class at final trial visit and a decrease in body weight of ≥5% from baseline, analysed using a hierarchal win ratio approach. Patients randomized to dapagliflozin were 34% more likely to have a favourable outcome than those randomized to placebo, however this benefit was driven predominantly by the effect on cardiometabolic components of the hierarchal composite with no significant benefit seen on the individual outcomes of cardiovascular death or heart failure hospitalization (although the trial was ultimately not powered for these outcomes).^39^

The Empagliflozin in Acute Myocardial Infarction (EMMY) trial investigated the effect of empagliflozin compared with placebo on NT‐proBNP concentrations in 476 patients within 72 h of a large acute MI (defined by creatine kinase >800 IU/L or troponin assay >10× upper limit of normal).^42^ The primary outcome was the change in NT‐proBNP from randomization to 26 weeks. Empagliflozin reduced NT‐proBNP by 15% (95% CI − 4.4 to −23.6%; *P* = 0.026) compared with placebo. Additionally, there were favourable placebo‐corrected changes in the secondary echocardiographic outcomes of LVEF (+1.5%), LVESV (−7.5 mL) and LVEDV (−9.7 mL).

The Impact of Dapagliflozin on Cardiac Function Following Anterior Myocardial Infarction in Non‐diabetic Patients (DACAMI) trial randomized 100 patients with anterior STEMIs and without T2DM to dapagliflozin or placebo for 12 weeks.^43^ The primary outcomes assessed were changes in NT‐proBNP levels and echocardiographic markers of remodelling. NT‐proBNP was reduced more in the dapagliflozin group, but only left ventricular mass index showed a significant difference in echocardiographic measures.^43^ The trial was powered for NT‐proBNP, and the shorter duration and limitations of echocardiography may explain the lack of observed remodelling benefits. Details of several other ongoing randomized placebo‐controlled trials of SGLT2 inhibitors in patients following acute MI are shown in *Table* *2*.

**Table 2:** Ongoing randomized trials of SGLT2 inhibitors in the post‐MI period

Trial	*n*	Population/design	Drug	Duration	Primary outcome	Status
EMPRESS‐MI (NCT05020704)	100	STEMI or NSTEMI, LVEF <45% Randomized, double‐blind	Empagliflozin	24 weeks	Change in LVESV index as measured by CMR	Active, completed recruitment
DAPAPROTECTOR (NCT05764057)	450	STEMI or NSTEMI, LVEF≤45% Randomized, double‐blind	Dapagliflozin	6 months	Change in LVEF and LAV as measured by echocardiography	Recruiting
NCT05050500	300	STEMI or NSTEMI and T2DM Randomized, double‐blind	Dapagliflozin	6 months	MACE, LVEF, LVEDV and LVESV as measured by CMR, post‐infarct angina, heart failure occurrence	Recruiting
NCT05045274	300	STEMI and LVEF<50% Randomized, double‐blind	Dapagliflozin	3 months	Improvement in LVEF (≥5%) as measured by echocardiography	Unknown
NCT06174753	256	Anterior STEMI Randomized, double‐blind	Dapagliflozin	7 days	Infarct size as measured by CMR	Not yet recruiting
PRESTIGE‐AMI (NCT04899479)	200	STEMI or NSTEMI, LVEF<50% or heart failure or both Randomized, open‐label	Not specified	6 months	Infarct size and change in LVESV as measured by CMR	Recruiting
DAPA‐AMI (NCT04717986)	188	STEMI and T2DM Randomized, double‐blind	Dapagliflozin	12 months	MACE	Completed
NCT05957887	120	STEMI Randomized, open‐label	Dapagliflozin	18 months	Global longitudinal strain as measured by echocardiography	Recruiting
NCT05305911	80	STEMI, LVEF <50%, infarct size >10% left ventricular mass and microvascular obstruction >10% infarct size Randomized, double‐blind	Dapagliflozin	6 months	Change in LVESV index and LVEDV index as measured by CMR	Recruiting
NCT06009874	80	STEMI Randomized, double‐blind	Dapagliflozin	3 months	Change in infarct size and NT‐proBNP	Recruiting
NCT04783870	60	STEMI or NSTEMI Randomized, double‐blind	Dapagliflozin	6 months	Change in LVEDV and LVESV as measured by CMR	Recruiting
NCT05335629	54	Anterior STEMI and T2DM Randomized, open‐label	Dapagliflozin	4 weeks	Effect on ST2	Completed

CMR, cardiovascular magnetic resonance; LAV, left atrial volume; LVEDV, left ventricular end‐diastolic volume; LVEF, left ventricular ejection fraction; LVESV, left ventricular end‐systolic volume; MACE, major adverse cardiovascular events; NSTEMI, non‐ST‐elevation myocardial infarction; NT‐proBNP, N‐terminal pro‐B‐type natriuretic peptide; ST2, suppression of tumourigenicity 2; STEMI, ST‐elevation myocardial infarction; T2DM, type 2 diabetes mellitus.

There are several important differences worth highlighting between EMMY, DACAMI, and EMPRESS‐MI. Firstly, EMPRESS‐MI is investigating LVESVI as the primary outcome using the gold‐standard technique of CMR rather than echocardiography, which will allow us to additionally investigate the effect of empagliflozin on other markers of cardiac remodelling including tissue characterization and infarct size estimation. Patients enrolled in EMPRESS‐MI had a higher risk of progressive adverse remodelling because of their greater degree of systolic dysfunction at baseline (EMPRESS‐MI inclusion LVEF <45% vs. mean LVEF approximately 50% in EMMY). Furthermore, we included patients with T2DM who have a greater risk of the development of chronic heart failure following acute MI as compared with those without T2DM, unlike the DAPA‐MI and DACAMI trials that excluded patients with T2DM.^44^ Other differences in the EMPRESS‐MI inclusion criteria will provide new data on the safety of initiation of an SGLT2 inhibitor following acute MI in patients with lower systolic blood pressure (>90 mmHg) and eGFR (>30 mL/min/1.73 m^2^) than in EMMY (blood pressure ≥100/70 mmHg and eGFR >45 mL/min/1.73 m^2^).

### Potential mechanisms of action of SGLT2 inhibitors post‐MI

**Figure 3:**
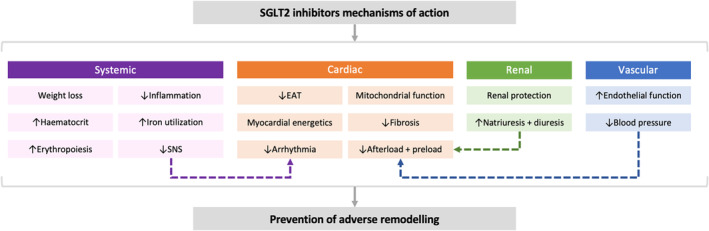
Mechanisms of action of SGLT2 inhibitors. Proposed mechanisms of benefit of SGLT2 inhibitors which could translate to prevention of adverse remodelling post‐MI. EAT, epicardial adipose tissue; SGLT2, sodium‐glucose cotransporter 2; SNS, sympathetic nervous system.

The mechanisms of action underlying the clinical benefits of SGLT2 inhibitors are not completely understood.^45^ However, some suggested mechanisms could theoretically contribute to a favourable effect on remodelling in the specific pathophysiological conditions arising following acute MI (*Figure* *3*).^46^ The multiparametric cardiac and kidney imaging data along with the extensive biomarker analyses of the EMPRESS‐MI trial will provide novel insights into the potential mechanisms of benefit of SGLT2 inhibition in high‐risk survivors of acute MI. These include the effect of empagliflozin on infarct size, intramyocardial haemorrhage and remote zone myocardial fibrosis along with circulating biomarkers of pro‐fibrotic processes, the relationship between SGLT2 inhibitors' effects on erythropoiesis, iron metabolism and ventricular remodelling, and the effect on biomarkers of inflammation (e.g., interleukin‐6, high sensitivity C‐reactive protein, and tissue necrosis factor‐α).^47^, ^48^, ^49^

### Rationale for key aspects of the design of EMPRESS‐MI

The dose of empagliflozin (10 mg once daily) in EMPRESS‐MI was based on the dose used in licensed indications and the clinical benefit and safety results seen with this dose in Empagliflozin Outcome Trial in Patients with Chronic Heart Failure with Reduced Ejection Fraction (EMPEROR‐Reduced) and Empagliflozin, Cardiovascular Outcomes, and Mortality in Type 2 Diabetes (EMPA‐REG OUTCOME).^50^, ^51^ The same dose was studied in the EMPACT‐MI trial.^40^

CMR is the gold standard method of assessment of left ventricular volumes and function. The primary outcome, LVESVI, is a major determinant of outcome post‐MI.^52^ Additionally, CMR allows assessment of myocardial viability, tissue characterization including estimation of infarct size, myocardial haemorrhage and myocardial iron content, myocardial fibrosis and regional dysfunction.^53^ Therefore, as well as providing data regarding the effect of empagliflozin on left ventricular volumes and function, EMPRESS‐MI will provide novel mechanistic insights into the direct myocardial effects of SGLT2 inhibition using state‐of‐art imaging techniques. Furthermore, a comprehensive series of circulating biomarker analyses will provide mechanistic insight into the effect of empagliflozin in our trial population.

The sample size calculation for the primary outcome was selected as it represents a minimally important difference.^9^, ^30^, ^54^, ^55^

Kidney dysfunction post‐MI is an adverse prognostic marker.^56^ The effects of SGLT2 inhibitors on kidney function post‐MI are uncertain. An additional novel aspect of this trial is the assessment of SGLT2 inhibition following acute MI on circulating biomarkers of kidney function and kidney tissue characteristics on CMR with kidney imaging.^57^

## Conclusions

Despite substantial therapeutic advances in the management of acute MI, the development of adverse left ventricular remodelling post‐MI and subsequent risk of heart failure remains a common occurrence. SGLT2 inhibitors improve outcomes in patients with chronic heart failure, and this is due, in part, to a prevention of adverse remodelling. SGLT2 inhibitors reduce heart failure events in patients at high risk following acute MI. Whether this is due to a remodelling benefit is, as yet, unknown. EMPRESS‐MI will provide novel multiparametric cardiac and kidney imaging and circulating biomarker data on the effect of empagliflozin in patients at high risk of heart failure following acute MI.

## Funding

The EMPRESS‐MI trial was funded by Boehringer Ingelheim. Drs Berry, Petrie, and McMurray are supported by a British Heart Foundation Centre of Research Excellence Grant RE/18/6/34217.

## Conflict of interest

Jaclyn Carberry reports that her employer, the University of Glasgow, has received research funding from Boehringer Ingelheim for her work relating to the EMPRESS‐MI trial. Ross Campbell has received consultancy honoraria from Bayer and for speaking honoraria from AstraZeneca. Pardeep S. Jhund reports grants from Boehringer Ingelheim, Analog Devices Inc., AstraZeneca, Roche Diagnostics; his employer, Glasgow University, has been remunerated for work on clinical trials with AstraZeneca, Novartis, Novo Nordisk, Bayer; Speaker fees and/or advisory board fees with AztraZeneca, Novartis, Boehringer Ingelheim; Lecture fees from AstraZeneca, Novartis, Inta Pharma, Sun Pharmaceuticals, ProAdwise; Director of Global Clinical Trial Partners Ltd. Ninian Lang received speaker fees from Roche, Pfizer, and Novartis, consultancy fees from Astra Zeneca and research grant support from Boehringer Ingelheim and Roche Diagnostics outside the submitted work. Matthew Lee's employer, the University of Glasgow, has received grant support from AstraZeneca and Boehringer Ingelheim. Patrick Mark reports personal fees from Astrazeneca, Astellas, GSK, grants from Boehringer Ingelheim, personal fees from Pharmacosmos, personal fees and non‐financial support from Napp, outside the submitted work. Alex McConnachie recognizes grant support from Diabetes UK. John JV McMurray has received payments through Glasgow University for work on clinical trials, consulting and other activities from Alnylam, Amgen, AstraZeneca, Bayer, Boehringer Ingelheim, BMS, Cardurion, Cytokinetics, Dal‐Cor, GSK, Ionis, KBP Biosciences, Novartis, Pfizer, Theracos; personal lecture fees from the Corpus, Abbott, Hikma, Sun Pharmaceuticals, Medscape/Heart.Org, Radcliffe Cardiology, Servier, Director, Global Clinical Trial Partners. Stuart Watkins reports honoraria from Abbott, AstraZeneca, Biosensors, Boston Scientific, Daiichi Sankyo, GE Healthcare, and ShockWave Medical and support for meeting attendance from Biosensors and Boston Scientific. Naveed Sattar recognizes grant support from AstraZeneca, Boehringer Ingelheim, Novartis, and Roche Diagnostics, has received consulting fees from Abbott Laboratories, Amgen, AstraZeneca, Boehringer Ingelheim, Eli Lilly, Hanmi Pharmaceuticals, Janssen, Marck Sharp & Dohme, Novartis, Novo Nordisk, Pfizer, Roche Diagnostics, and Sanofi, and has received payment for lectures or manuscript writing from Abbott Laboratories, AstraZeneca, Boehringer Ingelheim, Eli Lilly, Janssen, and Novo Nordisk. Paul Welsh reports grant income from Astrazeneca, Novartis, Roche Diagnostics, and Boehringer Ingelheim outside the submitted work and consultancy fees from Novo Nordisk and Benecol. Colin Berry is employed by the University of Glasgow which holds consultancy and research agreements for his work with Abbott Vascular, AstraZeneca, Auxilius Pharma, Boehringer Ingelheim, Causeway Therapeutics, Coroventis, Genentech, GSK, HeartFlow, Menarini, Neovasc, Novartis, Servier, Siemens Healthcare, and Valo Health. Mark C Petrie reported personal fees from AstraZeneca, Boehringer Ingelheim, NovoNordisk, Roche, Siemens, Takeda, New Amsterdam, Novartis, AbbVie, Pharmacosmos, and Vifor and grants from Boehringer Ingelheim, AstraZeneca, NovoNordisk, SQ Innovations, Roche, Novartis, and Pharmacosmos. Kieran F Docherty's employer, the University of Glasgow, has been remunerated by AstraZeneca for his work on clinical trials; he has received speaker fees from AstraZeneca, Boehringer Ingelheim, Pharmacosmos and Radcliffe Cardiology; has served on Advisory Boards for Us2.ai and Bayer AG; has served on a Clinical Endpoint Committee for Bayer AG; and has received research grant support (paid to his institution) from AstraZeneca, Roche, Novartis and Boehringer Ingelheim.
